# Enhanced Resistance to Fungal and Bacterial Diseases Due to Overexpression of BSR1, a Rice RLCK, in Sugarcane, Tomato, and Torenia

**DOI:** 10.3390/ijms24043644

**Published:** 2023-02-11

**Authors:** Satoru Maeda, Wataru Ackley, Naoki Yokotani, Katsutomo Sasaki, Norihiro Ohtsubo, Kenji Oda, Masaki Mori

**Affiliations:** 1 Institute of Agrobiological Sciences, NARO (NIAS), Tsukuba 305-8634, Japan; 2 Institute of Livestock and Grassland Science, NARO (NILGS), Nasushiobara 329-2793, Japan; 3 Research Institute for Biological Sciences, Okayama Prefectural Technology Center for Agriculture, Forestry, and Fisheries, Okayama 716-1241, Japan; 4 Institute of Vegetable and Floriculture Science, NARO (NIVFS), Tsukuba 305-0852, Japan

**Keywords:** *BSR1*, RLCK (receptor-like cytoplasmic kinase), disease resistance, *Sporisorium scitamineum*, *Rhizoctonia solani*, *Pseudomonas syringae*, sugarcane, rice, tomato, torenia

## Abstract

Sugarcane smut caused by *Sporisorium scitamineum* is one of the most devastating sugarcane diseases. Furthermore, *Rhizoctonia solani* causes severe diseases in various crops including rice, tomato, potato, sugar beet, tobacco, and torenia. However, effective disease-resistant genes against these pathogens have not been identified in target crops. Therefore, the transgenic approach can be used since conventional cross-breeding is not applicable. Herein, the overexpression of *BROAD-SPECTRUM RESISTANCE 1* (*BSR1*), a rice receptor-like cytoplasmic kinase, was conducted in sugarcane, tomato and torenia. *BSR1*-overexpressing tomatoes exhibited resistance to the bacteria *Pseudomonas syringae* pv. *tomato* DC3000 and the fungus *R. solani,* whereas *BSR1*-overexpressing torenia showed resistance to *R. solani* in the growth room. Additionally, *BSR1* overexpression conferred resistance to sugarcane smut in the greenhouse. These three *BSR1*-overexpressing crops exhibited normal growth and morphologies except in the case of exceedingly high levels of overexpression. These results indicate that *BSR1* overexpression is a simple and effective tool for conferring broad-spectrum disease resistance to many crops.

## 1. Introduction

Crop diseases are one factor that can cause severe damage to agricultural production. Therefore, crop protection strategies have been developed, including breeding disease-resistant varieties and using agrochemicals to minimize damage from crop diseases. Due to agrochemicals’ high cost and environmental impact, disease-resistant varieties are more desirable. The conventional method of generating disease-resistant varieties involves introducing resistance genes to the causative pathogen from resistant plants by cross-breeding. However, this method is limited to the pathogen species for which resistance genes have been identified.

Among the pathogens with a wide host range, the necrotrophic fungus *Rhizoctonia solani* causes serious diseases in hundreds of plant species. *R. solani* is a highly destructive pathogen that causes severe symptoms in the leaves, stems, and roots of diverse plant species, including rice [1], tomato [2,3], and torenia [4,5], leading to significant economic losses. To date, no cultivar has shown strong resistance to *R. solani*. Additionally, there are few reports of disease-resistant genes for *R. solani*, and it is difficult to breed varieties resistant to the fungus in many crops.

Sugarcane smut caused by the biotrophic fungus *Sporisorium scitamineum* is an important disease affecting sugarcane worldwide, leading to serious losses in productivity and profitability. However, there are no effective fungicides for sugarcane smut. Sugarcane is an economically important crop for sugar production. This disease degrades the yield and quality of the raw material for sugar production. Despite the worldwide loss of sugarcane yield and sucrose caused by *S. scitamineum*, limited information is available regarding its pathogenic mechanisms [6]. The disease is recognized by whip-like structures produced on the terminal meristem and side shoots of infected stalks [7]. However, few effective resistant genetic resources have been reported for this fungus. Furthermore, cultivated sugarcane is an interspecific hybrid polyploid with singularly complex genomes [8], making it difficult to efficiently breed resistant varieties against the disease. Additionally, cross-breeding is limited to specific regions because it flowers only in low-latitude regions. Therefore, it is difficult to breed sugarcane varieties resistant to smut through conventional breeding methods. Based on the above reasons, genetic engineering would be advantageous in breeding smut-resistant sugarcane. However, few genes that confer resistance to *S. scitamineum* have been identified in plants.

Previously, we identified *BROAD-SPECTRUM RESISTANCE 1* (*BSR1/OsRLCK278*) and *2* (*BSR2/CYP78A15*) by screening for resistance to the bacterial pathogen *Pseudomonas syringae* pv. *tomato* DC3000 (*Pst*DC3000) and the fungal pathogen *Colletotrichum higginsianum* using approximately 21,000 of the rice-FOX *Arabidopsis* lines [9,10]. Hence, *BSR1*-overexpressing (OX) *Arabidopsis* displays resistance to *Pst*DC3000 and *C. higginsianum.* Additionally, *BSR1* reportedly confers broad-spectrum resistance against the bacterial [*Xanthomonas oryzae pv. oryzae* (*Xoo*) and *Burkholderia glumae*] and fungal (*Pyricularia oryzae* and *Cochliobolus miyabeanus*) pathogens in rice [9,11]. Therefore, the introduction of *BSR1* using transgenic technology is expected to confer disease resistance to other gramineous and dicot crops.

The main objective of this study was to validate whether *BSR1* overexpression could confer disease resistance in tomatoes and the ornamental plant torenia, representing dicot crops, and sugarcane, representing monocot crops. *BSR1* was introduced into torenia because genetically modified (GM) ornamental crops, such as blue roses, are easily sold and more readily accepted by the general public than edible GM crops. First, *BSR1* was overexpressed in tomato, torenia, and sugarcane. Next, disease resistance tests against pathogens were conducted, gross morphology was observed in each crop, and some morphological traits were examined for practical use. As a result, *BSR1* overexpression successfully conferred disease resistance in three crops without detectable growth defects.

## 2. Results

### 2.1. BSR1 Overexpression Conferred Bacterial Pst DC3000 Resistance to Dicot Tomato

*BSR1* overexpression increases resistance to *Pst* DC3000 in *Arabidopsis* [9]. Since *Pst* DC3000 was originally a tomato pathogen, tomatoes overexpressing *BSR1* are expected to show resistance to the pathogen. Furthermore, the full-length cDNA of *BSR1* was previously inserted into the binary vector pBIG2113SF to generate *BSR1*-OX *Arabidopsis* [9]. This construct was used to transform the tomato cultivar ‘Micro-Tom’ by the *Rhizobium*-mediated method. Moreover, *BSR1* overexpression was confirmed in the independent transgenic tomato lines #12, #18, and #30 by quantitative real-time (qRT)-PCR (Figure S1a). The progenies were used for subsequent disease resistance assays.

**Figure 1:**
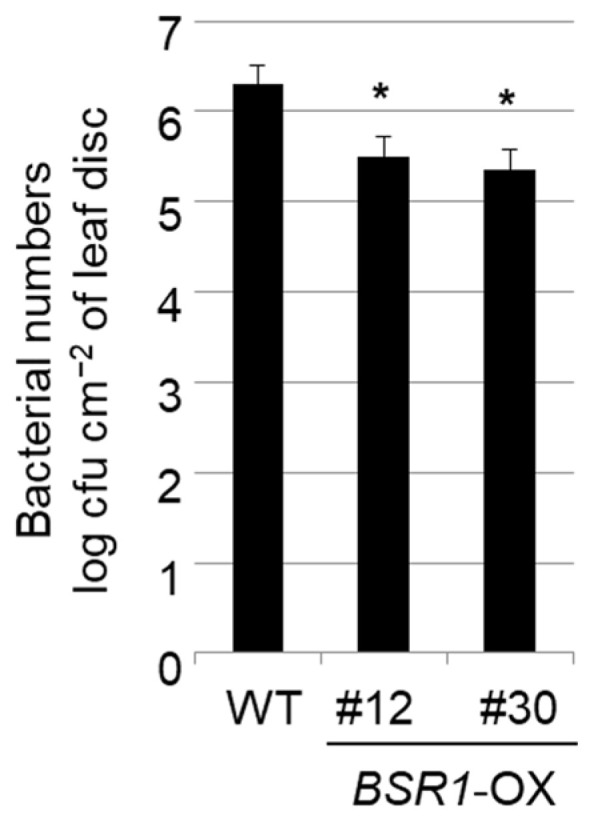
Disease resistance to the bacterial pathogen *Pst* DC3000 in *BSRI*-OX tomato. Tomato plants were inoculated with *Pst* DC3000 by the dipping method, and the number of bacteria present in sampled leaf discs was counted after 3 days. Error bars indicate standard deviations (*n* = 4–6). Asterisks indicate values significantly different from WT (*p* < 0.05; Dunnett’s test).

*BSR1*-OX and wild-type (WT) tomato plants were dip-inoculated with *Pst* DC3000. Subsequently, three days after inoculation, the bacterial number in leaf disks (1 cm^2^) collected from the inoculated plants was calculated to quantify the resistance. The average bacterial numbers in *BSR1*-OX tomato #12 and #30 lines were significantly reduced to approximately 1/6 and 1/9, respectively, compared with WT (Figure 1). Therefore, *BSR1* overexpression conferred resistance to *Pst* DC3000 in tomatoes.

### 2.2. BSR1 Overexpression Conferred Fungal R. solani Resistance to Arabidopsis and Tomato

**Figure 2:**
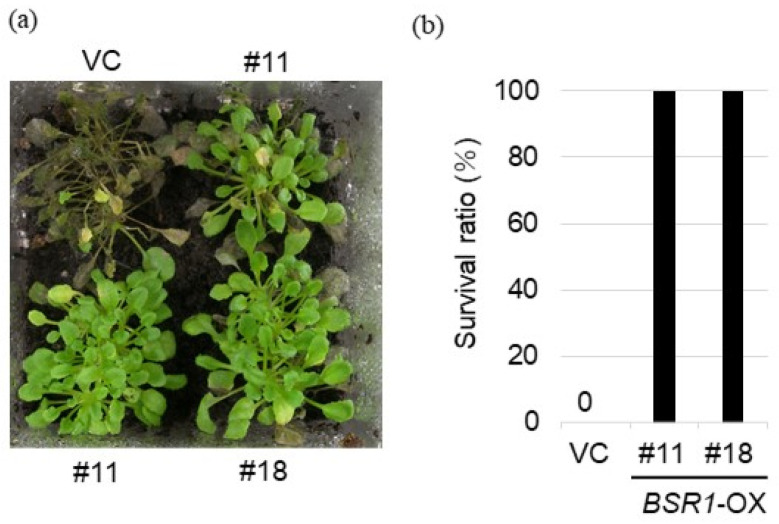
Disease resistance to the fungus *R. solani* in *BSRI*-OX *Arabidopsis*. Phenotypic response (**a**) and survival ratio (**b**) of *R. solani* isolate (MAFF243956; AG-1 IA). Using the soil inoculation method, 3-week-old plants of *BSRI*-OX and vector control (VC) *Arabidopsis* lines were inoculated with *R. solani*. The survival ratio (the number of surviving plants divided by that of the tested plants) was determined 33 days after inoculation. *n* = 6–12.

Next, we investigated whether *BSR1*-OX tomatoes were resistant to pathogenic fungi. We previously reported that *BSR1*-OX *Arabidopsis thaliana* does not tolerate *R. solani* infection in leaves [10]. However, in tomatoes, *R. solani* causes damping-off disease—a root disease. Therefore, in the present study, we first examined whether *BSR1*-OX *Arabidopsis* is resistant to *R. solani* root infection as a pilot experiment. Two *BSR1*-overexpressing *Arabidopsis* lines (#11 and #18) were subjected to root infection of *R. solani* isolate (MAFF243956; AG-1 IA). *BSR1*-OX *Arabidopsis* displayed a remarkable resistance to the root infection of *R. solani* (Figure 2).

**Figure 3:**
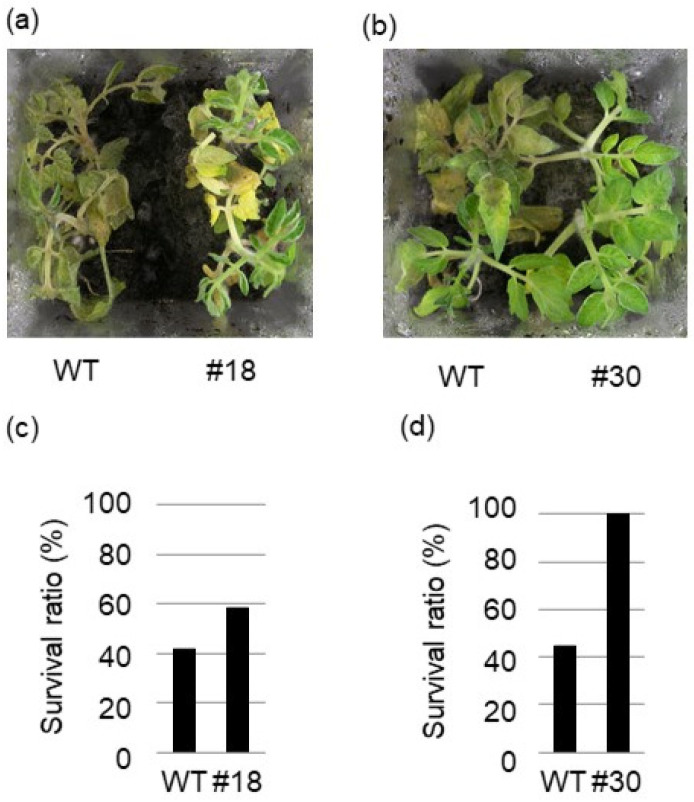
Disease resistance to the fungus *R. solani* in *BSRI*-OX tomato. Phenotypic response (**a**,**b**) and survival ratio (**c**,**d**) to *R. solani* isolate (MAFF235116; AG-4 IIIA) in the soil inoculation assay. The survival ratio (the number of surviving plants divided by that of the tested plants) was determined 6 days after inoculation. (**a**,**c**) *n* = 12, (**b**,**d**) *n* = 9. The tests were performed thrice, and similar results were obtained.

This result prompted us to proceed with the root infection experiment using tomatoes. For tomatoes, *R. solani* isolate (MAFF235116, AG-4 IIIA), whose pathogenicity is stronger than that of *R. solani* isolate (MAFF243956) [12], was used. *BSR1*-OX and WT tomato plants were inoculated with *R. solani* isolate using soil inoculation assay methods, as previously described [12]. As a result, more than half of the WT plants withered; however, more than half of the *BSR1*-OX #18 and #30 plants survived 6 days after inoculation (Figure 3a,b). The survival rates of *BSR1*-OX #18 and #30 (58% and 100%, respectively) were higher than those of the WT (42–44%) (Figure 3c,d). These data indicate that *BSR1* can confer resistance to *R. solani* in tomatoes and *Arabidopsis*. Hence, *BSR1* is expected to confer resistance to other dicot crops.

### 2.3. Resistance to R. solani in BSR1-OX Torenia

The soil-borne fungus *R. solani* causes devastating diseases in hundreds of plant species, including ornamental plants. Since *BSR1* overexpression confers resistance against *R. solani* in *Arabidopsis* and tomatoes, we investigated this effect in ornamental crops. We used torenia as a representative ornamental crop because it has a simple flower structure and is easy to culture and transform [13]. The *BSR1* expression vector, used for tomato transformation, was used for transforming the *Torenia fournieri* Lind. ‘Crown Violet’ to generate transgenic plants. As a result, overexpression of *BSR1* was confirmed in two independent T0 plants (Figure S1b).

**Figure 4:**
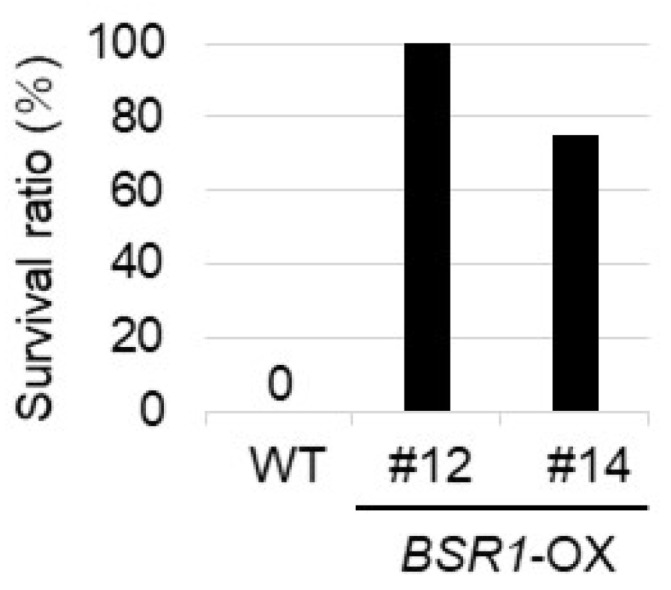
Disease resistance to *R. solani* in *BSRI*-OX torenia. Survival ratio of transgenic torenia against *R. solani* (MAFF235116: AG-4 IIIA) in soil inoculation assay. The survival ratio (number of surviving plants divided by that of the tested plants) was determined 5 days after inoculation. *n* = 4–5. The tests were performed thrice, and similar results were obtained.

*R. solani* resistance assay was performed using two *BSR1*-OX lines (#12 and #14). The survival ratios of *BSR1*-OX #12 and #14 lines (100% and 75%, respectively) were significantly higher than that of the WT line (0%) 5 days after inoculation (Figure 4). Therefore, *BSR1* overexpression conferred resistance to *R. solani* in torenia.

### 2.4. BSR1 Overexpression Conferred S. scitamineum Resistance to Monocot Sugarcane

The biotrophic fungal pathogen *S. scitamineum* causes sugarcane smut; however, the efficient breeding of varieties resistant to *S. scitamineum* remains a problem. Therefore, we examined whether *BSR1* confers *S. scitamineum* resistance in sugarcane. The cDNA of *BSR1* was inserted downstream of the constitutive 35S promoter with two 35S enhancers, and the construct was used to generate transgenic sugarcane lines using particle bombardment methods. After confirming cDNA insertion in the sugarcane genome by PCR, transgenic lines (T0) expressing BSR1 protein were screened by Western blot analysis. Lines 14 (lowly expressed), 30 (moderately expressed), and 35 (highly expressed) were used for the following experiments (Figure S1c).

**Figure 5:**
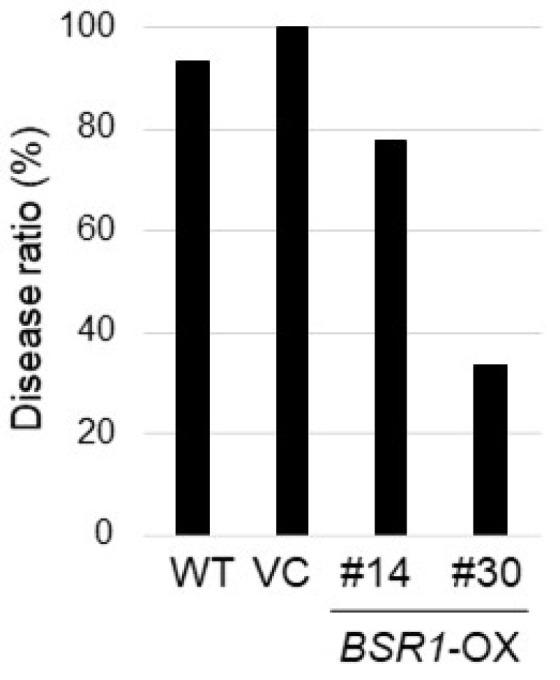
Disease resistance to the fungus *S. scitamineum* in *BSRI*-OX *sugarcane*. The disease ratio of transgenic sugarcane exposed to *S. scitamineum* axillary buds of *BSRI*-OX, vector control (VC), and WT plants were inoculated with *S. scitamineran* via needle injection. The disease ratio (number of plants with black whip-like structures divided by that of the tested plants) was determined 150 days after inoculation. *n* = 3–15. The tests were performed thrice, and similar results were obtained.

Furthermore, axillary buds in WT and transgenic plants were inoculated with 10^4^ spores/mL *S. scitamineum* via needle injection. When *S. scitamineum* infects susceptible sugarcane plants, the fungus causes black whip-like structures in the infected plants (Figure S2) [6,14]. Therefore, the number of plants with black whip-like structures was counted, and the disease ratio was calculated. The disease ratios of *BSR1*-OX #14 and #30 (78% and 33%, respectively) were lower than those of the vector control (VC; 100%) and WT (93%) 150 days after inoculation (Figure 5). Additionally, the disease ratios of *BSR1*-OX #30 and #35 (64% and 27%, respectively) were lower than that of the vector control (100%) 130 days after inoculation in the other experiment (Figure S3). As described above, three *BSR1*-OX lines were resistant to *S. scitamineum*. Lastly, BSR1 protein expression levels correlate roughly with disease resistance levels (Figure 5, Figures S1c and S3).

### 2.5. Morphological Traits and Growth of BSR1-OX Plants

To breed and use the *BSR1* gene, it is important that the gene does not affect other traits moreover disease resistance. Therefore, the growth and morphogenesis of *BSR1* overexpressing plants were observed.

**Figure 6:**
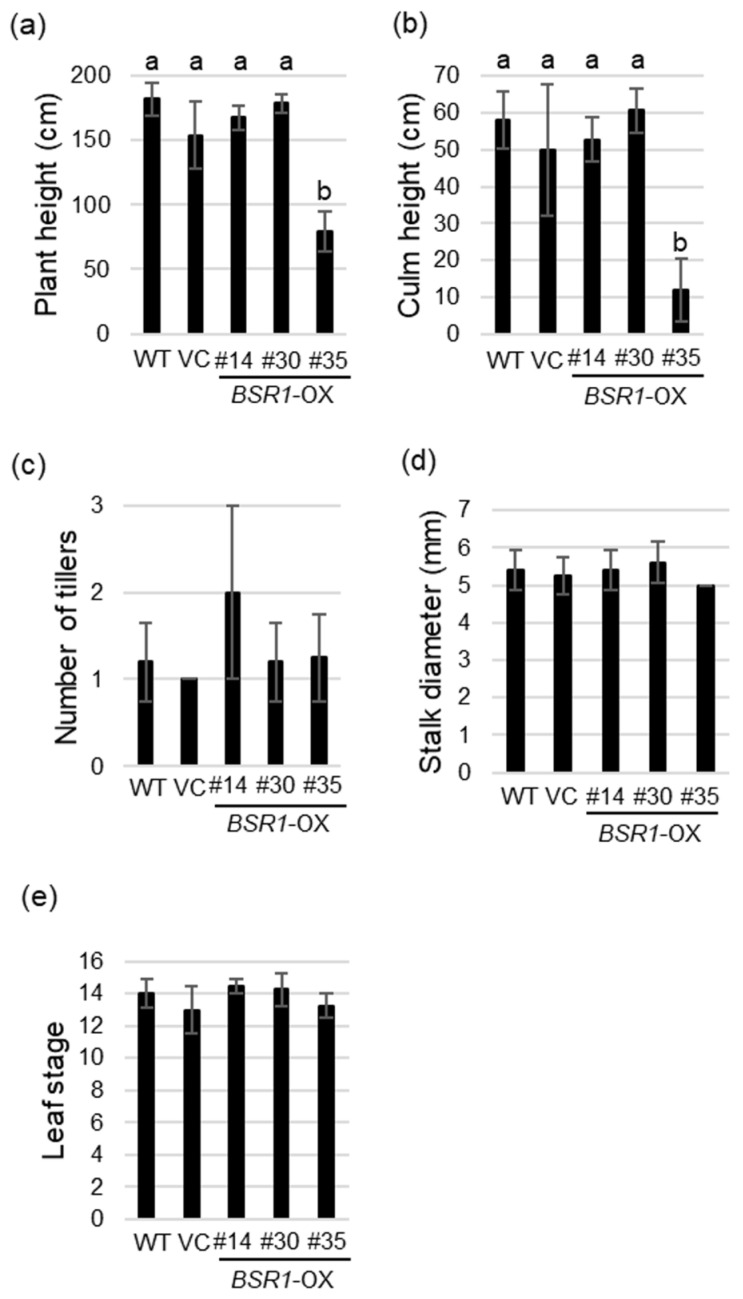
Morphological traits in *BSRI*-OX sugarcane. Comparison of (**a**) plant height, (**b**) clum height, (**c**) the number of tillers, (**d**) stalk diameter, and (**e**) leaf stage among WT, vector control (VC), and three *BSRI*-OX lines 180 days after transplanting. Different letters indicate significant differences according to Tukey’s test (*p* < 0.05). Error bars represent standard deviation. *n* = 4–5.

Plant height, culm height, number of tillers, and stalk diameter of three *BSR1*-OX lines, WT, and VC plants were measured 180 days after transplanting because such agronomic traits affect yield in sugarcane. Additionally, the leaf stage was examined to evaluate its effect on growth. Plant and culm heights of *BSR1*-OX #14 and #30 lines were not significantly different from those of the WT and VC lines (Figure 6a,b and Figure S4); however, those of #35 line, with the highest BSR1 expression level, were significantly lower (Figure 6a,b and Figure S1c). Therefore, a high accumulation of the BSR1 protein would have repressed normal growth. Regarding the number of tillers and stalk diameter, the values of the three *BSR1*-OX lines were not significantly different from those of the WT and VC (Figure 6c,d). These data suggest that *BSR1*-OX #14 and #30 lines had no negative effects on yield traits. Additionally, no significant difference was detected in the leaf stage among the WT, VC, and three *BSR1*-OX lines (Figure 6e). These results indicate that *BSR1* does not affect growth and yield traits if its expression level is adequately controlled.

**Figure 7:**
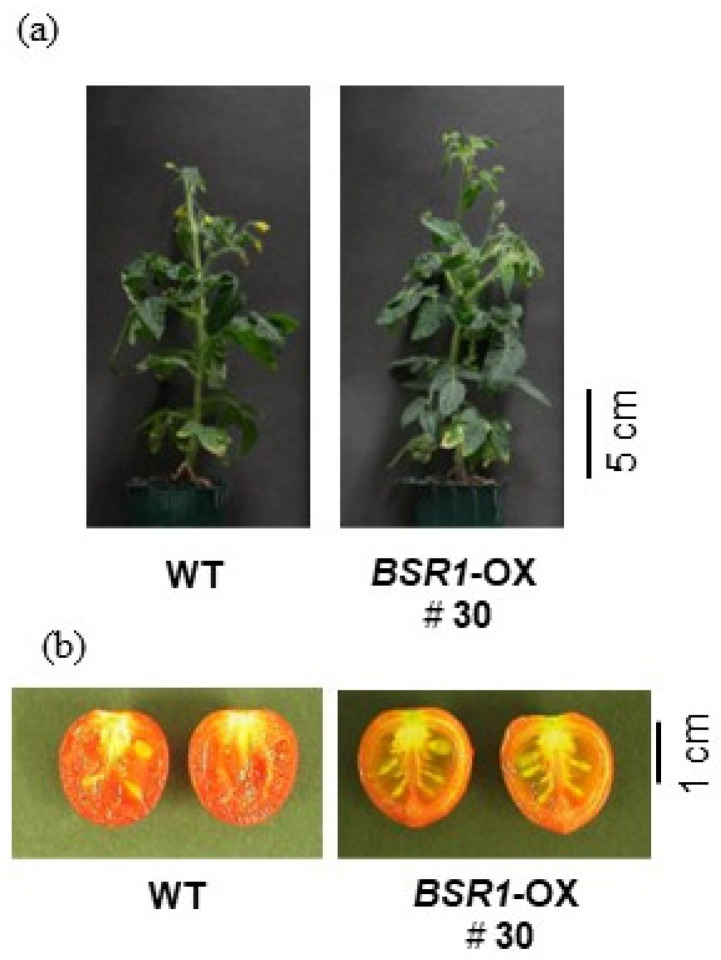
Morphological traits in *BSRI*-OX tomato. (**a**) Gross morphology of *BSRI*-OX (line #30) and WT tomato plants grown in the greenhouse 63 days after sowing. (**b**) Fruits of *BSRI*-OX (line #30) and WT plants.

**Figure 8:**
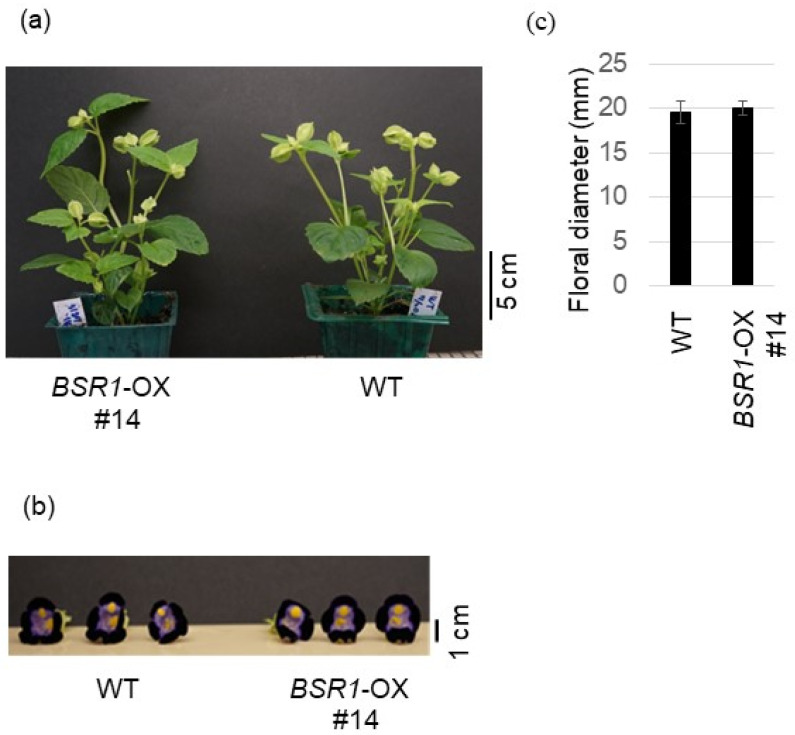
Morphological traits in *BSRI*-OX torenia. (**a**) Gross morphology of *BSRI*-OX (line #14) and WT torenia plants grown in a growth room under long-day conditions (16 h light and 8 h dark) at 25 °C 66 days after propagation by cutting. (**b**,**c**) Flowers (**b**) and floral diameter (**c**) of *BSRI*-OX (line #14) and WT plants 3 days after flowering. Error bars indicate standard deviation (*n* = 4).

In tomatoes, the gross plant morphology and agronomically important fruit size of *BSR1* overexpressing typical lines were similar to those of the WT tomatoes (Figure 7). Additionally, in torenia, the gross plant morphology and agronomically important flower size of *BSR1* overexpressing typical lines were similar to those of the WT torenia (Figure 8). Overall, with appropriate *BSR1* expression, adverse effects on growth and morphology were not observed in the tested transgenic crops (tomato, torenia, and sugarcane).

## 3. Discussion

### 3.1. Broad-Spectrum Disease Resistance by BSR1 Overexpression in Sugarcane, Tomato, and Torenia

*BSR1* overexpression in *A. thaliana*, a model dicot plant, confers resistance to the bacterial pathogen *Pst* DC3000 and the fungal pathogen *C. higginnsianum* [9]. Additionally, *BSR1* overexpressing *Arabidopsis* plants showed *R. solani* underground resistance (roots). To validate such resistance in tomato, a representative dicot crop, *BSR1* was overexpressed in tomato cv. Micro-Tom. *BSR1*-OX tomatoes showed resistance to *Pst* DC3000 and *R. solani*. Furthermore, overexpression of *BSR1* in torenia, a representative ornamental dicot crop, also confers resistance to *R. solani*. When *BSR1* was overexpressed in sugarcane, a monocot crop, it showed resistance to *S. scitamineum*, a pathogenic fungus that causes sugarcane smut, the most important disease in sugarcane. Therefore, overexpression of the rice *BSR1* gene would be a powerful tool to confer broad-spectrum disease resistance in dicot and monocot crops, including ornamental plants.

Plant pathogens are often divided into biotrophs, hemibiotrophs, and necrotrophs, according to their parasitic types. *S. scitamineum, Pst* DC3000, and *R. solani* used in this study were classified as biotrophs, hemibiotrophs, and necrotrophs, respectively. *BSR1* overexpression conferred resistance to these three pathogens. The disease resistance mechanism of BSR1 is expected to enhance pattern-triggered immunity (PTI), elicited in the very early stages of infection when the host cells are still alive. Together, all three pathogens used in this study may have been in the biotrophic phase at the early stages of infection, when *BSR1* overexpression exerted disease resistance.

### 3.2. Broad-Spectrum Disease-Resistant Mechanism by BSR1 Overexpression in Sugarcane, Tomato, and Torenia

*BSR1* encodes a receptor-like cytoplasmic kinase, OsRLCK278, which is classified into the same RLCK-VII subfamily as *Arabidopsis* BIK1 [9]. We reported that BSR1 plays a role in chitin-, peptidoglycan-, and lipopolysaccharide-triggered defense responses and promotes reactive oxygen species (ROS) production [15,16]. In addition, OsCERK1, a hub-RLK, is necessary for enhanced PTI response and blast resistance by *BSR1* overexpression [17]. Hence, BSR1 plays a major regulatory role in various MAMP-induced PTI responses under OsCERK1 in rice. Furthermore, since dicot *Arabidopsis* has CERK1 (an orthologue of OsCERK1) and BIK1 and PBL kinases homologous to BSR1, sugarcane, tomato, and torenia would have proteins and PTI systems similar to those of rice. Therefore, we speculated that *BSR1* overexpression would enhance the PTI system by hijacking the innate PTI system in each crop.

The closest homologues to *BSR1* in *Arabidopsis* are PBL19 and PBL20, which belong to the RLCK-VII subfamily [18]. Additionally, members of the RLCK-VII-4 subgroup, including PBL19 and PBL20, are involved in chitin-triggered MPK3/6, MPK4 activation, ROS production, defense gene expression, and redundantly confer basal resistance to the bacterial pathogen [19,20]. Moreover, Li et al. [21] reported that after induction by chitin, a fraction of PBL19 protein translocates into the nucleus via the N-terminal nuclear localization sequence and induces transcriptional self-amplification primarily by WRKY8. Moreover, increased PBL19 proteins interact with EDS1 and promote antifungal immunity. Although BSR1′s role has not been elucidated compared to PBL19, a common phenomenon between BSR1 and PBL19 is involved in chitin-triggered immunity and broad disease resistance against bacterial and fungal pathogens, suggesting that overproduced BSR1 protein function is similar to PBL19 or PBL19-like proteins, therefore potentiating their effects in dicot *Arabidopsis*, tomato, torenia, and monocot sugarcane.

Although *BSR1* has been overexpressed by the constitutive CaMV*35S* promoter in the whole plant in *Arabidopsis*, the resulting *BSR1*-OX *Arabidopsis* plants did not show resistance to *R. solani* in their leaves [10]. However, in the present study, *BSR1*-OX *Arabidopsis, BSR1*-OX tomato, and *BSR1*-OX torenia showed resistance against *R. solani* in the root. Furthermore, *Arabidopsis* RLCKs, such as PBL19 and BIK1, interact with other proteins to function properly [21,22]. Notably, PBL19 is involved in immunity against *Verticillium dahliae*—a soil-borne fungal pathogen [21]. Therefore, an interactant of BSR1 may be present in roots but absent in leaves, leading to root-specific *R. solani* resistance in *BSR1*-OX *Arabidopsis.*

There are many reports of chitin-induced immunity in the rice RLCK-VII subfamily besides BSR1. Furthermore, the rice orthologue of PBL27 (OsRLCK185) [23,24,25,26], and the rice homologues of BIK1 (OsRLCK57, OsRLCK107, OsRLCK118, and OsRLCK176) [27,28], are involved in chitin-triggered signaling. *BSR1* appears to be the only RLCK-VII subfamily gene reported to be overexpressed in multiple heterologous plants and confers resistance to a wide range of pathogens, as observed in this study. Since this phenotypic disease resistance trait is unique to *BSR1*, a specialized working mechanism would exist compared with other RLCK-VII proteins involved in chitin-triggered immunity.

Moreover, if the expression levels were appropriate, the growth and morphologies of *BSR1*-OX plants would be similar to those of WT and vector control plants in tomato, *Arabidopsis*, torenia, and sugarcane, as observed in this study. Considering the trade-off between immunity and growth [29], PTI may have a regulatory function, preventing unnecessary immunity activation. Negative regulators, such as the phosphatase CIPP1 and ubiquitin E3 ligases (PUB12 and 13), involved in PTI, have been found in *Arabidopsis* [30,31]. In rice, OsCERK1, OsRLCK57, OsRLCK107, and OsRLCK185 have been identified as ubiquitinated proteins after chitin induction [32]. Based on the above reports, any negative regulator which prevents prolonged activation of MAMP-induced immunity would be present in the immune mechanism of *BSR1* overexpression. Moreover, the PBL19′s function is regulated by translocation into the nucleus [21]; therefore, the BSR1′s function, related to the trade-off between immunity and growth, may be regulated by nuclear translocation in each host plant. Nevertheless, the negative regulatory function of BSR1 should be elucidated to understand the reasons for the normal morphology and growth of *BSR1*-OX plants observed in this study.

### 3.3. Toward Applications for Generating Broad-Spectrum Disease-Resistant Crops

If disease-resistant genes enhance plants’ defense capacity, plant pathogens’ negative impact on agricultural productivity will be reduced without using chemicals, such as fungicides and bactericides. However, to date, the most commonly used disease-resistant genes for breeding in agriculture are *R* genes which induce effector-triggered immunity, and few genes involved in PTI have been used. *BSR1* is one of the candidate genes involved in PTI that can be used to breed disease-resistant crops. Although detailed disease resistance mechanisms are required, our results indicate that *BSR1* overexpression could be applied to different crops to confer broad-spectrum disease resistance.

## 4. Materials and Methods

### 4.1. Plant Materials and Culture

*Solanum lycopersicum* L. cv. Micro-Tom was used as the WT tomato. Micro-Tom (accession No. TOMJPF00001) was provided by the University of Tsukuba, Tsukuba Plant Innovation Research Center, through the National Bio-Resource Project of the AMED, Tsukuba, Japan. The WT and transgenic tomatoes were grown as previously described [12]. Moreover, *Torenia fournieri* Lind. ‘Crown Violet’ was used as a WT torenia material. The violet-flowered CrV cultivar selected from F1 hybrid seeds of ‘Crown Mix’ (Sakata Seed Co., Yokohama, Japan) was kindly provided by Dr. Ryutaro Aida (Institute of Vegetable and Floriculture Science, NARO, Tsukuba, Japan). One vigorously growing F1 hybrid plant was propagated vegetatively via herbaceous cutting by Dr. Ryutaro Aida and used as the experimental line in this study. The WT and transgenic torenia plants were cultured and maintained as previously described [13].

A Japanese commercial sugarcane cultivar, “KRFo93-1” (a Saccharum spp. hybrid), developed at the Kyushu Okinawa Agricultural Research Center (KARC), NARO, Japan, was used as the WT sugarcane. The WT and transgenic sugarcane were grown as follows. First, the plant stem internodes were cut and germinated under a 14/10 h light/dark regime and humidified conditions at 30 °C for 4 to 7 days. Next, the germinated nodes were used in the tests. Afterwards, for growth and morphological observations, the germinated nodes were transplanted into the soil (Bonsol No. 2, Sumitomo Chemical Co., Ltd., Osaka, Japan) and grown in a greenhouse under a natural day length for 180 days.

### 4.2. Plasmid Construction and Transformation

To generate transgenic tomato and torenia plants that overexpressed *BSR1,* the full-length cDNA fragment of *BSR1* was inserted downstream of the CaMV 35S promoter at the *Sfi*I sites of the recombinant binary vector pBIG2113SF [9] and the resulting plasmid pBIG2113SF-BSR1 was introduced into *Rhizobium radiobacter* strain EHA105. This construct was employed to generate lines of transgenic tomato and torenia plants using the *Rhizobium*-mediated method [13,33,34]. In torenia, T0 and WT plants were propagated through cuttings and used for the subsequent experiments.

For sugarcane, the *Hin*dIII-*Eco*RI fragment cut from pBIG2113SF-BSR1 was introduced into the multi-cloning site of the pBC-SK plasmid (Stratagene, San Diego, CA, USA), and the resulting plasmid pBC-BSR1 was used for the transformation. Additionally, transgenic sugarcane plants were produced using a PDS-1000/He helium-driven biolistic device (Bio-Rad, Hercules, CA, USA) according to the method described by Nagai et al. [35]. The basic parameters of the bombardment were the same as for the Italian ryegrass transformation [36]. All culture media used were based on the Murashige and Skoog (MS) medium [37] containing 3% (w v^−1^) sucrose, adjusted to pH 5.8, and solidified with 0.25% (w v^−1^) Gelrite (Wako, Osaka, Japan). For callus induction, apical meristems of glasshouse-grown plants were aseptically isolated and cultured on a callus induction medium containing 0.25 mg L^−1^ benzyladenine, 4 mg L^−1^ 2,4-dichlorophenoxyacetic acid, and N6 vitamins [38] in the dark at 25 °C. Induced calli were subcultured monthly. Subsequently, the calli, approximately 3–4 months old, were divided into small pieces and arranged in a circle (3 cm in diameter) on a high-osmotic pretreatment medium containing 0.25 M sorbitol and 0.25 M mannitol and incubated for 24 h in the dark at 25 °C. Next, the calli were co-bombarded with the plasmids pAcH1 [39] and pBC-BSR1. Notably, pAcH1 and pBC-BSR1 were used to express hygromycin phosphotransferase and *BSR1*, respectively. Afterwards, the bombarded calli were cultured in the dark at 25 °C. After two days, the calli were transferred to a selection medium supplemented with 150 mg L^−1^ hygromycin and subcultured to a fresh selection medium every two weeks. Moreover, hygromycin-resistant calli grown on the selection medium were cultured on a phytohormone-free regeneration medium containing 3 g L^−1^ activated charcoal under continuous fluorescent light (40 µmol m^−2^ s^−1^) at 25 °C until green-rooted shoots were regenerated from the resistant calli. Next, the rooted plants were established in soil pods and grown in a glasshouse at 28 °C. Subsequently, the transformation was evaluated using PCR analysis with Ampdirect^®^ Plus (Shimazu, Kyoto, Japan) according to the manufacturer’s instructions in a ProFlex™ PCR System (Thermo Fisher Scientific, Waltham, MA, USA). The primer sets, 5′-CGCATAACAGCGGTCATTGACTGGAGC-3′ and 5′-GCTGGGGCGTCGGTTTCCACTATCGG-3′were used to detect the 375-bp fragment of pAcH1, and 5′-AGGTGAGGTTGCACTCTGCT-3′ and 5′-ACATAGATGACACCGCGCGCGATAATTTATC-3′ to detect the 506-bp fragment of pBC-BSR1.

### 4.3. RNA Extraction and Quantitative Real-Time -PCR Analysis

Total RNA was extracted and purified from tomato and torenia leaves using Sepasol-RNA Super G reagent (Nacalai Tesque, Kyoto, Japan), according to the manufacturer’s protocol. Next, first-strand cDNAs were synthesized, and qRT-PCR analysis was performed as previously described [12]. Afterwards, the *BSR1* transcript levels were normalized to those of the endogenous tomato and torenia reference genes. The primers used for qRT-PCR are listed in Table S1.

### 4.4. Protein Extraction and Western Blot Analysis

Plant protein was extracted from sugarcane leaves and immuno-detected according to a previously described method [40]. Next, custom-made rabbit polyclonal anti-BSR1 (Biogate, Gifu, Japan) was used to detect the BSR1 protein, as described by Sugano et al. [41].

### 4.5. Pathogens and Pathogen Cultures

The bacteria *Pseudomonas syringae* pv. *tomato* DC3000 (*Pst* DC3000) was cultured using a previously described protocol [9]. The fungus *R. solani* was cultured on PDA agar plates (Nissui, Tokyo, Japan) at 28 °C under dark conditions for 3 days and used for disease resistance tests. The spores of the fungus *S. scitamineum* were provided by Shin Irei (Okinawa Prefectural Agricultural Research Center, Japan).

### 4.6. Bacterial and Fungal Pathogen Resistance Assay in Arabidopsis, Tomato, Torenia, and Sugarcane

The bacterial pathogen assay for *Pst* DC3000 in tomatoes was performed as previously described [12]. Additionally, the soil inoculation assay of *R. solani* in *Arabidopsis*, tomato, and torenia was performed as previously described [12,13].

The inoculation assay of *S. scitamineum* in sugarcane was performed as follows. First, the germinated nodes were used for the tests. The plant stem internodes of WT, vector control, and three *BSR1*-OX sugarcane lines were cut and germinated under a 14/10 h light/dark regime and humidified conditions at 30 °C for 4 to 7 days. Next, the germinated axillary buds in the nodes were inoculated with 10^4^ spores/mL *S. scitamineum* by needle injection. Afterwards, the internodes with-infected axillary buds were transplanted into the soil (Bonsol No, Sumitomo Chemical Co., Ltd., Osaka, Japan) and grown in a greenhouse under natural day length. Lastly, the number of plants with the disease symptom, a black whip-like structure (Figure S2), was counted 130 or 150 days after inoculation, and the disease ratio (plant number with the symptom/inoculated plant number) was calculated.

## Data Availability

Data are contained within the article.

## Supplementary Materials

The following supporting information can be downloaded at: https://www.mdpi.com/article/10.3390/ijms24043644/s1.

## References

[1] Hane J.K., Anderson J.P., Williams A.H., Sperschneider J., Singh K.B. (2014). Genome sequencing and comparative genomics of the broad host-range pathogen *Rhizoctonia solani* AG8. PLoS Genet..

[2] Gondal A.S., Rauf A., Naz F. (2019). Anastomosis Groups of *Rhizoctonia solani* associated with tomato foot rot in Pothohar Region of Pakistan. Sci. Rep..

[3] Bartz F.E., Cubeta M.A., Toda T., Naito S., Ivors K.L. (2010). An In Planta Method for Assessing the Role of Basidiospores in Rhizoctonia Foliar Disease of Tomato. Plant Dis..

[4] Chen C.X., Wu Y.F., Gong H.H., Lin Y.J., Chen C.Y. (2021). First Report of Binucleate *Rhizoctonia* AG-L Causing Root and Stem Rot of Wishbone Flower (*Torenia fournieri*) in Taiwan. Plant Dis..

[5] Jiang S.B., Yang Q.Y., Lin B.R., Zhang J.X., Shen H.F., Pu X.M., Sun D.Y., Bai Y.B., Tang Z.Q. (2022). Occurrence of Root and Stem Rot Caused by *Rhizoctonia solani* AG-4 HGI on *Torenia fournieri* in China. Plant Dis..

[6] Que Y., Xu L., Wu Q., Liu Y., Ling H., Zhang Y., Guo J., Su Y., Chen J., Wang S. (2014). Genome sequencing of *Sporisorium scitamineum* provides insights into the pathogenic mechanisms of sugarcane smut. BMC Genom..

[7] Comstock J.C., Ferreira S.A., Tew T.L. (1983). Hawaii’s approach to control of sugarcane smut. Plant Dis..

[8] Zhang J., Zhang X., Tang H., Zhang Q., Hua X., Ma X., Zhu F., Jones T., Zhu X., Bowers J. (2018). Allele-defined genome of the autopolyploid sugarcane *Saccharum spontaneum* L.. Nat. Genet..

[9] Dubouzet J.G., Maeda S., Sugano S., Ohtake M., Hayashi N., Ichikawa T., Kondou Y., Kuroda H., Horii Y., Matsui M. (2011). Screening for resistance against Pseudomonas syringae in rice-FOX Arabidopsis lines identified a putative receptor-like cytoplasmic kinase gene that confers resistance to major bacterial and fungal pathogens in Arabidopsis and rice. Plant Biotechnol. J..

[10] Maeda S., Dubouzet J.G., Kondou Y., Jikumaru Y., Seo S., Oda K., Matsui M., Hirochika H., Mori M. (2019). The rice CYP78A gene BSR2 confers resistance to *Rhizoctonia solani* and affects seed size and growth in Arabidopsis and rice. Sci. Rep..

[11] Maeda S., Hayashi N., Sasaya T., Mori M. (2016). Overexpression of BSR1 confers broad-spectrum resistance against two bacterial diseases and two major fungal diseases in rice. Breed. Sci..

[12] Maeda S., Yokotani N., Oda K., Mori M. (2020). Enhanced resistance to fungal and bacterial diseases in tomato and Arabidopsis expressing BSR2 from rice. Plant Cell Rep..

[13] Maeda S., Sasaki K., Kaku H., Kanda Y., Ohtsubo N., Mori M. (2022). Overexpression of Rice *BSR2* Confers Disease Resistance and Induces Enlarged Flowers in *Torenia fournieri* Lind. Int. J. Mol. Sci..

[14] Carvalho G., Quecine M., Longatto D., Peters L., Almeida J., Shyton T., Silva M., Crestana G., Creste S., Monteiro-Vitorello C. (2016). Sporisorium scitamineum colonisation of sugarcane genotypes susceptible and resistant to smut revealed by GFP-tagged strains. Ann. Appl. Biol..

[15] Kanda Y., Yokotani N., Maeda S., Nishizawa Y., Kamakura T., Mori M. (2017). The receptor-like cytoplasmic kinase BSR1 mediates chitin-induced defense signaling in rice cells. Biosci. Biotechnol. Biochem..

[16] Kanda Y., Nakagawa H., Nishizawa Y., Kamakura T., Mori M. (2019). Broad-Spectrum Disease Resistance Conferred by the Overexpression of Rice RLCK BSR1 Results from an Enhanced Immune Response to Multiple MAMPs. Int. J. Mol. Sci..

[17] Kanda Y., Nishizawa Y., Kamakura T., Mori M. (2020). Overexpressed *BSR1*-Mediated Enhancement of Disease Resistance Depends on the MAMP-Recognition System. Int. J. Mol. Sci..

[18] Shiu S., Karlowski W., Pan R., Tzeng Y., Mayer K., Li W. (2004). Comparative analysis of the receptor-like kinase family in Arabidopsis and rice. Plant Cell.

[19] Rao S., Zhou Z., Miao P., Bi G., Hu M., Wu Y., Feng F., Zhang X., Zhou J. (2018). Roles of Receptor-Like Cytoplasmic Kinase VII Members in Pattern-Triggered Immune Signaling. Plant Physiol..

[20] Bi G., Zhou Z., Wang W., Li L., Rao S., Wu Y., Zhang X., Menke F.L.H., Chen S., Zhou J.M. (2018). Receptor-Like Cytoplasmic Kinases Directly Link Diverse Pattern Recognition Receptors to the Activation of Mitogen-Activated Protein Kinase Cascades in Arabidopsis. Plant Cell.

[21] Li Y., Xue J., Wang F.Z., Huang X., Gong B.Q., Tao Y., Shen W., Tao K., Yao N., Xiao S. (2022). Plasma membrane-nucleo-cytoplasmic coordination of a receptor-like cytoplasmic kinase promotes EDS1-dependent plant immunity. Nat. Plants.

[22] DeFalco T.A., Zipfel C. (2021). Molecular mechanisms of early plant pattern-triggered immune signaling. Mol. Cell.

[23] Yamaguchi K., Yamada K., Ishikawa K., Yoshimura S., Hayashi N., Uchihashi K., Ishihama N., Kishi-Kaboshi M., Takahashi A., Tsuge S. (2013). A receptor-like cytoplasmic kinase targeted by a plant pathogen effector is directly phosphorylated by the chitin receptor and mediates rice immunity. Cell Host Microbe.

[24] Yamaguchi K., Yoshimura Y., Nakagawa S., Mezaki H., Yoshimura S., Kawasaki T. (2019). OsDRE2 contributes to chitin-triggered response through its interaction with OsRLCK185. Biosci. Biotechnol. Biochem..

[25] Wang C., Wang G., Zhang C., Zhu P., Dai H., Yu N., He Z., Xu L., Wang E. (2017). OsCERK1-Mediated Chitin Perception and Immune Signaling Requires Receptor-like Cytoplasmic Kinase 185 to Activate an MAPK Cascade in Rice. Mol. Plant.

[26] Yamada K., Yamaguchi K., Yoshimura S., Terauchi A., Kawasaki T. (2017). Conservation of Chitin-Induced MAPK Signaling Pathways in Rice and Arabidopsis. Plant Cell Physiol..

[27] Ao Y., Li Z., Feng D., Xiong F., Liu J., Li J.F., Wang M., Wang J., Liu B., Wang H.B. (2014). OsCERK1 and OsRLCK176 play important roles in peptidoglycan and chitin signaling in rice innate immunity. Plant J..

[28] Li Z., Ao Y., Feng D., Liu J., Wang J., Wang H.B., Liu B. (2017). OsRLCK 57, OsRLCK107 and OsRLCK118 Positively Regulate Chitin- and PGN-Induced Immunity in Rice. Rice.

[29] Huot B., Yao J., Montgomery B.L., He S.Y. (2014). Growth-defense tradeoffs in plants: A balancing act to optimise fitness. Mol. Plant.

[30] Liu J., Liu B., Chen S., Gong B.Q., Chen L., Zhou Q., Xiong F., Wang M., Feng D., Li J.F. (2018). A Tyrosine Phosphorylation Cycle Regulates Fungal Activation of a Plant Receptor Ser/Thr Kinase. Cell Host Microbe.

[31] Yamaguchi K., Mezaki H., Fujiwara M., Hara Y., Kawasaki T. (2017). *Arabidopsis ubiquitin* ligase PUB12 interacts with and negatively regulates Chitin Elicitor Receptor Kinase 1 (CERK1). PLoS ONE.

[32] Chen X.L., Xie X., Wu L., Liu C., Zeng L., Zhou X., Luo F., Wang G.L., Liu W. (2018). Proteomic Analysis of Ubiquitinated Proteins in Rice. Front. Plant Sci..

[33] Aida R., Shibata M. (1995). Agrobacterium-Mediated Transformation of Torenia (*Torenia Fournieri*). Breed. Sci..

[34] Aida R. (2012). A protocol for transformation of Torenia. Methods Mol. Biol..

[35] Nagai K., Mori Y., Ishikawa S., Furuta T., Gamuyao R., Niimi Y., Hobo T., Fukuda M., Kojima M., Takebayashi Y. (2020). Antagonistic regulation of the gibberellic acid response during stem growth in rice. Nature.

[36] Takahashi W., Oishi H., Ebina M., Takamizo T., Komatsu T. (2002). Production of transgenic Italian ryegrass (*Lolium multiflorum* Lam.) via microprojectile bombardment of embryogenic calli. Plant Biotechnol..

[37] Murashige T., Skoog F. (1962). A revised medium for rapid growth and bio assays with tobacco tissue cultures. Physiol. Plant.

[38] Chu C.C., Wang C.C., Sun C.S., Hsu C., Yin K.C., Chu C.Y., Bi F.Y. (1975). Establishment of an efficient medium for anther culture of rice through comparative experiments on nitrogen-sources. Sci. Sin..

[39] Spangenberg G., Wang Z.Y., Wu X.L., Nagel J., Iglesias V.A., Potrykus I. (1995). Transgenic Tall Fescue (*Festuca arundinacea*) and Red Fescue (*F. rubra*) Plants from Microprojectile Bombardment of Embryogenic Suspension Cells. J. Plant Physiol..

[40] Matsushita A., Inoue H., Goto S., Nakayama A., Sugano S., Hayashi N., Takatsuji H. (2013). Nuclear ubiquitin proteasome degradation affects WRKY45 function in the rice defense program. Plant J..

[41] Sugano S., Maeda S., Hayashi N., Kajiwara H., Inoue H., Jiang C.J., Takatsuji H., Mori M. (2018). Tyrosine phosphorylation of a receptor-like cytoplasmic kinase, BSR1, plays a crucial role in resistance to multiple pathogens in rice. Plant J..

