# Supplementary material for: Enhanced Resistance to Fungal and Bacterial Diseases Due to Overexpression of BSR1, a Rice RLCK, in Sugarcane, Tomato, and Torenia
Source: Int J Mol Sci. 2023 Feb 11;24(4):3644. doi: 10.3390/ijms24043644 (PMC9965303; doi:10.3390/ijms24043644)
Supplement: Supplementary file 1 [file ijms-24-03644-s001.zip › ijms-2189268-supplementary.pdf]

Table S1. Primers used for quantitative real-time (qRT)-PCR.

Genes	Forward	Reverse
LeACT	TGGTCGATCCACCGGTATTGTG	AATGGCATGTGGAAGGGCATAC
TfACT3	AAGATATGCATTGGAGTTGTGAGTG	TCCAAGCTAAGGTAGCAAAACGA
BSR1	AGGTGAGGTTGCACTCTGCT	CCAAGAATCCACCAACTCGT

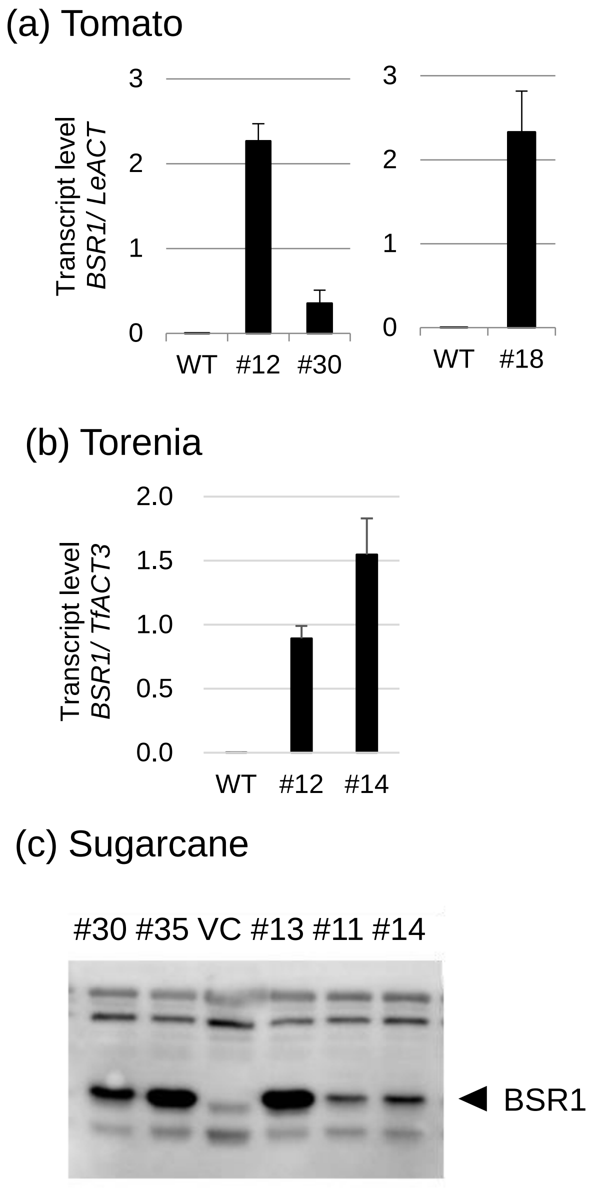

Figure S1. Transcript or protein levels of BSR1 in *BSR1*-OX lines.
(a) *BSR1* transcript levels in WT and three *BSR1*-OX transgenic tomato lines were determined using qRT-PCR. *BSR1* transcript levels were normalised to the endogenous actin gene *LeACT*. Error bars indicate standard deviations (left: $n = 3$, right: $n = 2–3$).
(b) *BSR1* transcript levels in WT and two *BSR1*-OX transgenic torenia lines were determined using qRT-PCR. *BSR1* transcript levels were normalised to the endogenous actin gene *TfACT3*. Error bars indicate standard deviations ($n = 3–4$).
(c) The levels of accumulated BSR1 protein in vector control (VC) and *BSR1*-OX transgenic sugarcane lines determined by Western blot analysis using an anti-BSR1 antibody. Black arrowhead, BSR1 protein (44.7 kDa).

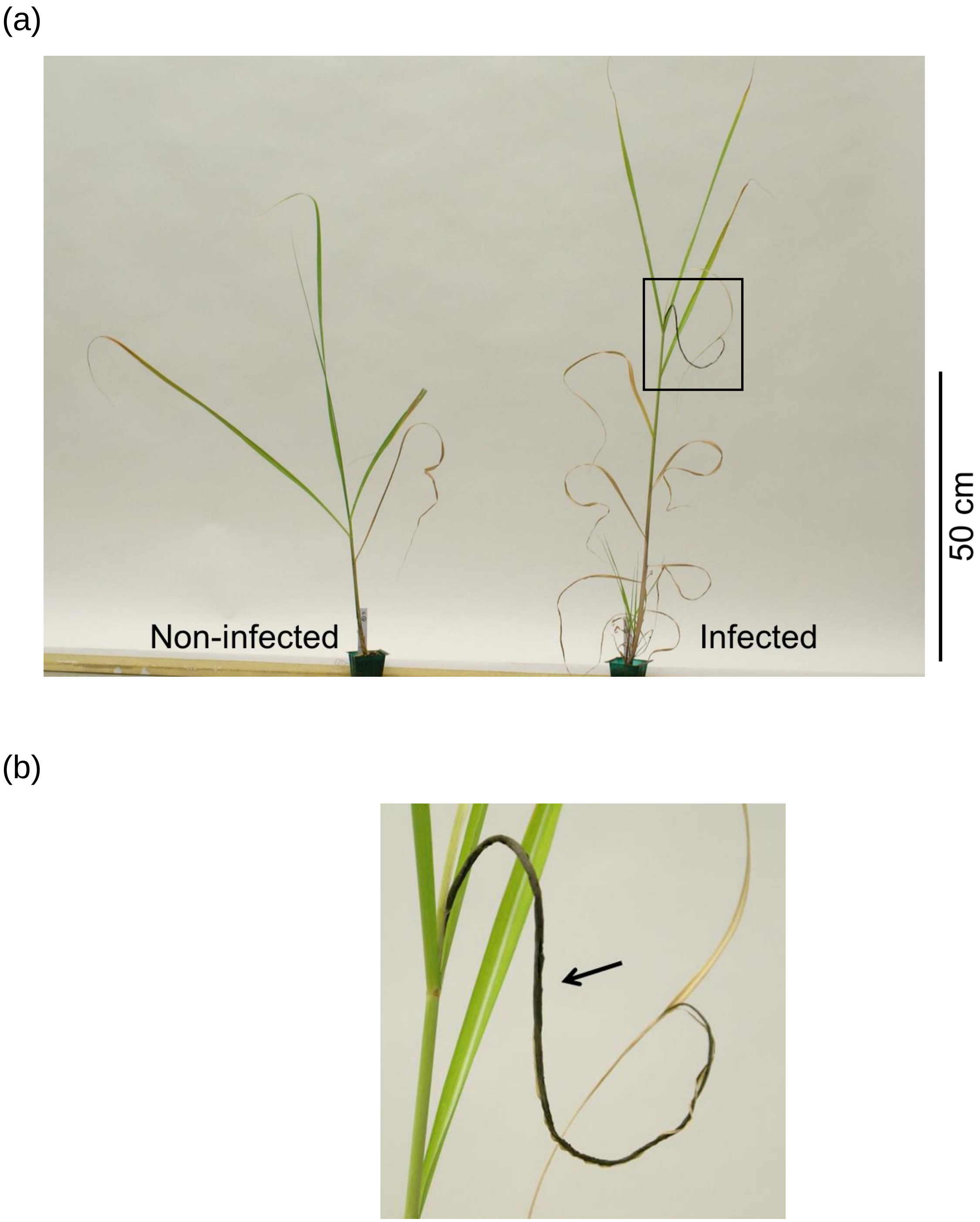

Figure S2. A black whip-like structure characteristic of sugarcane smut disease. (a) Gross morphology of non-infected and infected sugarcane plants 98 days after inoculation with *S. scitamineum*. (b) Close-up of the boxed region in (a). A black whip-like structure is indicated by an arrow.

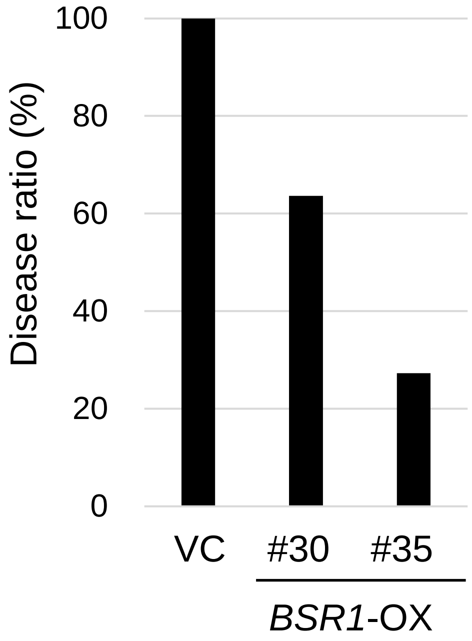

Figure S3. Disease resistance to the fungus *S. scitamineum* in *BSR1*-OX #30 and #35 sugarcane lines.

The disease ratio of transgenic sugarcane to *S. scitamineum* axillary buds of *BSR1*-OX and vector control (VC) plants were inoculated with *S. scitamineum* via needle injection. The disease ratio (number of plants with black whip-like structures divided by that of the tested plants) was determined 130 days after inoculation. *n* = 5–6. The tests were performed thrice, and similar results were obtained.

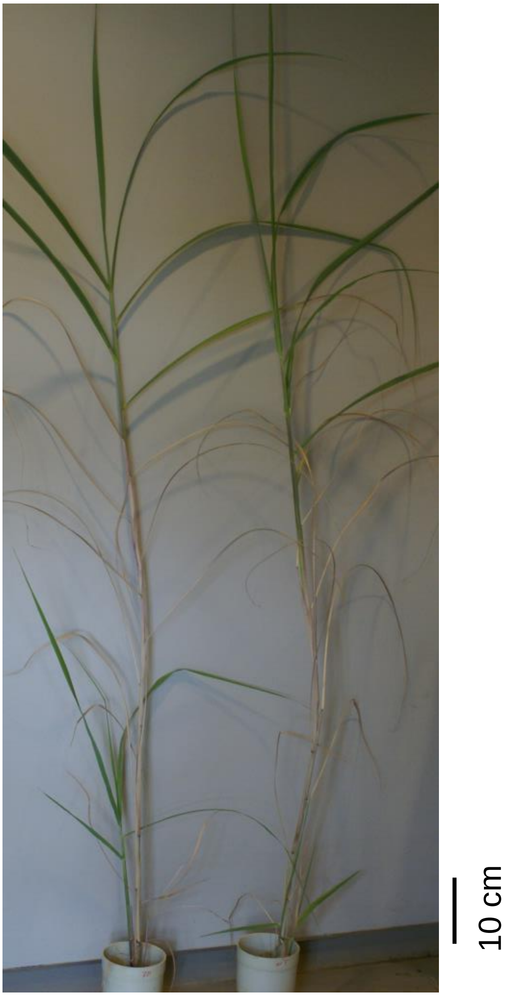

BSR1-OX WT
#30

Figure S4. Gross morphology of *BSR1*-OX sugarcane. *BSR1*-OX (line #30) and WT sugarcane plants were grown in the greenhouse 200 days after planting.
